# Targeting lysine demethylase 5 (*KDM5*) in mantle cell lymphoma

**DOI:** 10.1038/s41408-024-00999-8

**Published:** 2024-02-13

**Authors:** Danmei Xu, Findlay Bewicke-Copley, Karina Close, Jessica Okosun, Robert Peter Gale, Jane Apperley, David M. Weinstock, Hans-Guido Wendel, Jude Fitzgibbon

**Affiliations:** 1 Centre for Genomics and Computational Biology, Barts Cancer Institute, Queen Mary University of London, Charterhouse Sq, London, UK; 2 Centre for Haemato-Oncology, Barts Cancer Institute, Queen Mary University of London, Charterhouse Sq, London, UK; 3 Centre for Haematology, Imperial College London, Hammersmith Hospital, Du Cane Road, London, UK; 4 Department of Medical Oncology, Dana-Farber Cancer Institute, Boston, MA USA; 5 Memorial Sloan-Kettering Cancer Center, Cancer Biology & Genetics, New York, NY 10065 USA; 6 Present Address: Oxford Cancer and Haematology centre, Churchill Hospital, Oxford University Hospitals NHS Foundation Trust, Oxford, OX3 7LE UK; 7 Present Address: Merck and Co., Rahway, NJ USA

**Keywords:** Preclinical research, Targeted therapies

**TO THE EDITOR:**

The prognosis of advanced mantle cell lymphoma (MCL) is poor [1]. Analyses of genomic and epi-genomic landscapes indicate a critical role for the DNA methylome and its interplay with other genomic complexities including copy number alterations (CNAs) and breakage–fusion-bridge cycles [2, 3]. Unlike genomic mutations, the epigenome of cancer cells can be modified by therapeutic intervention [4].

Mutations in epigenetic regulators including *KMT2D*, *CREBBP*, and *EZH2* are common in B-cell lymphomas. Specifically, the mutation frequency of *KMT2D*, a histone methyltransferase in MCL is about 20% and is associated with a poor prognosis [5, 6]. These *loss-of-function* mutations decrease H3K4me1/me2 deposition and alter gene expressions. Effects of these mutations have only been modelled in germinal centre (GC) lymphomas and are difficult to target therapeutically [7–9]. Normal levels of H3K4 methylation are maintained by the KDM5 de-methylase family. *KDM5*, a component of the epigenetic repressor complex, removes the active transcriptional mark H3K4me3. Targeting *KDM5* and restoring epigenomic balance might improve the prognosis of lymphomas dependent on epigenetic dys-regulation as we described in GC-lymphomas [10, 11].

**Fig. 1:**
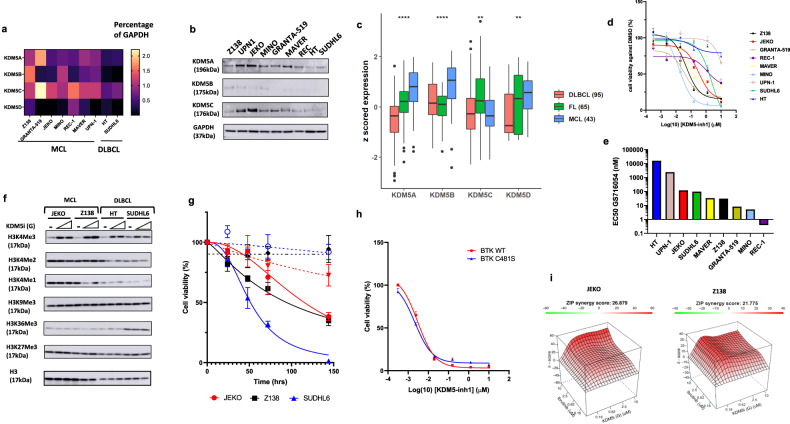
Targeting KDM5 demonstrates efficacy in killing MCL cells and can overcome Ibrutinib resistance. **a** *KDM5A-D* gene expression was measured by qRT-PCR in 7 MCL and 2 DLBCL cell lines, normalized to the expression of *GAPDH* (*n* = 3). **b** KDM5A-C protein expressions detected by Western blot. **c** *KDM5A-D* gene expression in MCL (*n* = 43), FL (*n* = 65), and DLBCL (*n* = 95) patient cohorts (GSE132929) were analysed (**P < 0.01; ****P < 0.0001). **d** Cell viability following 6 days exposure to DMSO or 6 dose concentrations of GS716054 ranging from 0.00031 to 10 µM (Gilead), determined by Cell Titer-Glo assays. **e** Bar chart showing the EC_50_ values of GS716054 on MCL and DLBCL cell lines. **f** Z138 and JEKO cells were exposed to DMSO, 1 or 5 µM GS716054 for 72 h, the histone marks were determined by Western blot accordingly. H3K4me3/me2/me1 (KDM5), H3K9me3/K36me3(KDM4), H3K27me3(KDM6). HT and SUDHL6 were included as controls. **g** Cell viability of JEKO, Z138, and SUDHL6 cells were determined following treatment with DMSO, 1 µM GS716054 (solid line) or JQKD82 (dot line) for 24, 48, 72 and 144 h. **h** Cell viability was assessed and compared in MINO cell lines stably expressing either WT BTK or BTK C481S mutant, following treatment as described at (**d**) (Triplicated, *P* > 0.05). **i** Viable JEKO and Z138 cells were analysed following treatment with increasing dosing of GS716054 for 6 days, alongside increasing concentrations of Ibrutinib for 3 days. ZIP synergy scores were calculated using Synergy Finder through 3 independent experiments. Representative 3D synergistic plot is shown here with a Synergy Score. Score > 10 indicates significant synergy.

*KDM5A-D* is rarely mutated in cancers but could be a potential therapy target based on upregulation in several cancers and function as a driver of drug-resistance [12]. KDM5 proteins are not detectable in B-cells from healthy individuals by immune blotting [13]. Whilst *KDM5A* and *KDM5B* are constitutively expressed isoforms, *KDM5C* and *KDM5D* are known X- and Y- chromosome-linked paralogs [7]. We determined the expression of these isoforms in MCL using cell lines and subject-derived samples. All *KDM5* isoforms are expressed in MCL cell lines (*n* = 7; Fig. 1a, b) including *KMT2D* mutated JEKO (R5225C) and Granta-519 (A1598V) cells, and in most resistant and sensitive diffuse large B- cell lymphoma (DLBCL) cell lines, to KDM5 inhibitors, HT and SUDHL6 [10]. Analyses of a human mature B-cell lymphoid dataset (GSE132929) indicate *KDM5A* and *KDM5B* are over-expressed in MCL subject samples compared with DLBCLs and follicular lymphomas (FLs) (Fig. 1c) [14]. Heterozygous *KMT2D* mutations were detected in 2 of 7 MCL cell lines. However, both mutated and unmutated MCL cell lines were sensitive to GS716054, a KDM5-inhibitor [10, 15], with an EC_50_ as low as 5.17 nM in MINO cells (Fig. 1d, e). Five of seven MCL cell lines showed greater sensitivity to GS716054 than SUDHL6, the most sensitive DLBCL cell line [10]. The MINO and REC cell lines with *TP53* mutations were also the most sensitive (Fig. 1d). Incubation of MCL cells with GS716054 increases global H3K4me3 levels at 1 and 5 μM, an effect stronger compared with incubation with JQKD82, another KDM5 inhibitor (Figs. 1f, g; S1a, b) [13]. Both effects were detectable at 24 h persisting over 6 days (Fig. S1c).

Granta-519 and Z138, ibrutinib-resistant cell lines, are sensitive to GS716054 (Figs. 1h; S2a, b) [16, 17]. The ibrutinib-resistant MINO cell line with Bruton Tyrosine Kinase (BTK) C481S mutation is as sensitive to GS716054 as the wild-type ibrutinib-sensitive parental cell line (Figs. 1i; S2c) [16]. These data suggest GS716054 acts independently of BTK signalling. Although JEKO cells have intermediate sensitivity to GS716054 and ibrutinib, a synergistic effect is evident with an average ZIP synergy score of 23.53 (Figs. 1e, j; S2b; S2d). In most MCL cell lines, there is a synergy found between GS716054 and ibrutinib (Fig. S2e).

The impact of *KMT2D* mutations in haematological and solid cancers varies and the precise mechanism(s) and downstream mediator(s) are debatable [7]. *BCL2* over-expression is seen in most patients with FLs, reflecting the occurrence of the t(14;18) translocation. We reported *BCL2* down-regulation in GC lymphoma models following treatment with a *KDM5*-inhibitor [10]. This effect was not observed in MCL cell lines (Fig. S1d).

**Fig. 2:**
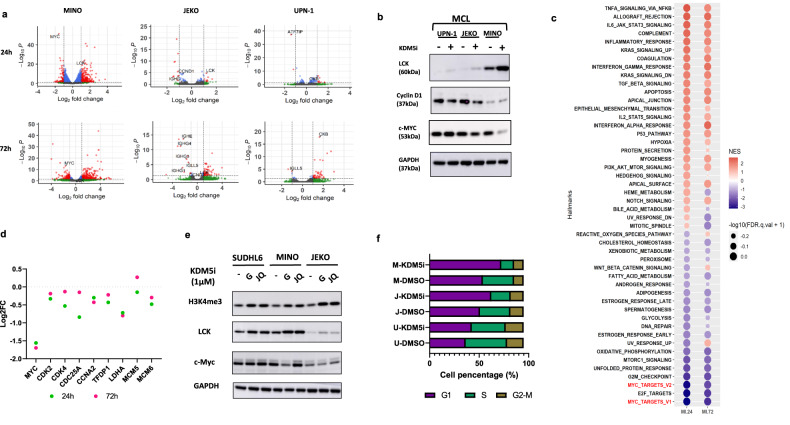
KDM5-inhibition regulates MYC target genes and associating cell cycle changes. **a** Volcano plots indicate differentially expressed (DE) genes in MINO, JEKO, and UPN-1 cells treated with 1 µM GS716054 for 24 and 72 h, with significant genes highlighted in red. **b** Western blotting to validate the protein expressions of LCK, c-MYC, and CyclinD1 following 1 µM GS716054. **c** RNA seq of MINO cells treated with 1 µM GS716054 or DMSO control for 24 or 72 h. GSEA for transcriptional hallmarks was shown by a bubble plot where the size of the bubbles indicates significance and normalized enrichment score (NES) indicates the strength of the enrichment. Each time points from triplicated RNA samples. **d** Dot plot showing the dynamic changes of a range of representative MYC target genes following 1 µM GS716054 or DMSO control for 24 or 72 h in MINO cells. Log2FC represents expression fold changes against DMSO control. **e** SUDHL6, MINO, and JEKO cells were exposed to DMSO, 1 µM GS716054, or JQKD82 for 72 h. Western blot showing the expression levels of indicated histone marks and proteins. **f** MINO, JEKO, and UPN-1 cells were treated with DMSO or 1 µM GS716054 for 72 h. Cell cycle profiles were analysed by flow cytometry.

To interrogate mechanism(s) underlying the efficacy of GS716054 by targeting KDM5 in MCL, we performed RNA-Seq on 3 MCL lines, UPN-1 (insensitive to GS716054), JEKO (intermediate sensitivity) and MINO (sensitive) cell lines following 24 or 72 h incubation with 1μM GS716054 or DMSO control (Figs. 2a; S3a, b). Significantly greater number of differentially expressed (DE) genes were detected in MINO compared with UPN-1 and JEKO cells paralleling their sensitivity profiles (Fig. S4a; GSE243395). Most DE genes were up-regulated (Fig. S4b). Expression changes were most striking at 24 vs. 72 h in MINO cells (2486 vs. 623 DE genes).

Lymphocyte-specific tyrosine kinase (*LCK)*, a Src family member, is involved in the activation and cytokine production of T-cell receptor signaling. Endogenous levels of LCK varied between cell lines. Interestingly, we found *LCK* activation in MCL cell lines, more in the MINO and SU-DHL6 (DLBCL) cell lines, the most sensitive to GS716054 (Figs. 2a, b; S5).

Expression of *MYC* and its target genes decreased after GS716054 treatment of MCL cells. This was most striking in the MINO cell line (Figs. 2b–d; S5a). *MYC* gene sets were the most strongly down-regulated class across the hallmark gene sets from the Molecular Signatures Database (MSigDB)) correlating with the transcript levels of representative *MYC* target genes (Figs. 2c, d and S6). Hence, downstream mediators of KDM5 inhibition in MCL may be distinct from those in GC lymphomas. *MYC* over-expression is associated with a poor prognosis in mature B-cell neoplasms but has proven difficult to target. Consequently, most strategies focus on reducing transcription or translation. A study in plasma cell myeloma showed *MYC* expression was suppressed by KDM5A-inhibition via an RNA polymerase II-dependent mechanism [13]. Transcriptomic profiling of cells from patients with MCL, those with *TP53* mutation, and *MYC* over-expression have the worst prognosis [3]. Our data suggest the potential role of a *KDM5*-inhibitor in these challenging cases.

Transcriptomic analyses comparing ibrutinib-sensitive and -resistant MCL cell lines indicate suppression of a *MYC* gene signature only in ibrutinib-sensitive MCL cell lines. *MYC* knock-down with RNA interference (RNAi) inhibited cell growth in ibrutinib-sensitive and -resistant MCL cell lines implicating *MYC* expression in ibrutinib resistance [18]. *LCK* induction and MYC suppression were stable regardless of expressing *BTK*^WT^ or *BTK*^C481S^ suggesting KDM5-inhibition associated suppression of the MYC pathway is independent of *BCR*-signalling (Fig. S5b), highlighting the potential of KDM5 inhibitor as an alternative therapy approach in people with MCL resistant to ibrutinib.

Our ChIP-Seq data indicated *LCK* transcriptional loci overlays GS716054-induced H3K4me3 activation in GC lymphomas [10]. JQKD82 treatment which induces H3K4 tri-methylation comparably to GS716054 but is less cytotoxic to MCL cells, paralleling induced *LCK* expression but has only a modest effect on MYC expression, indicating increased LCK expression results from increased H3K4 tri-methylation whereas *MYC* regulates death of MCL cells after KDM5-inhibition (Fig. 2e). These data suggest a non-catalytic function of KDM5 family may also contribute to the cytotoxic effects of GS716054 on MCL cells.

GS716054 induces G_1_ cell cycle arrest but not apoptosis in JEKO cells suggesting growth inhibition mediated by KDM5-inhibition results predominately from cell cycle arrest (Figs. 2f, S7a). This effect was not found with JQKD82 treatment (Fig. S7b). Gene-set enrichment analysis (GSEA) showed expressions of cell-cycle genes were reduced following the treatment by GS716054 (Fig. S7c). This correlates with sensitivity to KDM5-inhibition and paralleling our finding of marked G_1_ arrest in MINO cells compared to minimal changes in GS716054 resistant cell line UPN-1 (Figs. 2f; S7c).

*TP53* and *MYC* mutations are strongly associated with therapy resistance and adverse prognosis in MCL [1, 3]. We found MINO and REC cells with *TP53* mutations were highly sensitive to *KDM5*-inhibitors which can overcome ibrutinib resistance. These data suggest a possible role for *KDM5*-inhibitors in advanced MCL. It would be imperative to test and evaluate the in vivo efficacy of GS716054 in an MCL patient-derived xenograft (PDX) model, particularly in the context of *KMT2D*, *TP53* and *MYC* aberrations as well as BTK inhibitor resistance. Confirmation of our in vitro observations would further define the molecular groups of MCL patients most likely to benefit and support evaluation in early-phase clinical trials.

### Supplementary information

supplementary material

supplementary figures

Supplemental Table
