# Supplementary material for: Targeting lysine demethylase 5 (KDM5) in mantle cell lymphoma
Source: Blood Cancer J. 2024 Feb 13;14(1):29. doi: 10.1038/s41408-024-00999-8 (PMC10864367; doi:10.1038/s41408-024-00999-8)
Supplement: Supplementary file 1 — supplementary material [file 41408_2024_999_MOESM1_ESM.docx]

**Supplement**

**Material and Methods**

**Cell culture**

Human mantle cell lymphoma cell lines (Z138, JEKO, GRANTA-519, REC-1, MAVER, MINO, UPN-1) and the MINO BTK WT and C481S cell lines were cultured as described in a 5% CO2, 37°C humidified incubator using RPMI-1640 supplemented with 20% FBS, 1% L-glutamine and 1% Pen-Strep, except Z-138 and Granta-519 which were cultured in IMEM or DMEM respectively with 10% FBS, 1% L-glutamine and 1% Pen-Strap (10, 16). Cells were checked to ensure absence of mycoplasma. *KMT2D* mutations have been validated by Sanger sequencing.

**Proliferation assays**

Viable cells (3000/well) were seeded in 100 μl growth media in triplicate in 96-well plates for 24 h before treating with DMSO or 6 concentrations of *KDM5-*inhibitors (GS716054 or JQKD82) diluted 8-fold (0.0003-10 μM) in 100 μl of growth medium for indicated time points. Viable cell numbers were determined using Cell Titre-Glo luminescent cell viability assay (Promega). Viability percentage was determined by normalizing luminescent readouts to DMSO controls.

**Western blots**

Whole cell lysates were extracted and lysed as described (10). Briefly, cells were lysed in RIPA buffer (Invitrogen) supplemented phosphatase and Complete ULTRA protease inhibitor cocktails (Roche). Lysates were quantified by Protein Assay Reagent (ThermoFisher). To assess histone mark levels, 2.5µg of protein was loaded and resolved in 4- 12% Bis-Tris gels (NuPAGE), followed by blotting via the iBlotTM transfer device (InVitrogen). Most primary antibodies used are described (10), Additional antibodies are from Abcam: LCK (32149), Cyclin D1 (134175), c-Myc (32072). Secondary antibodies are from DAKO. Protein bands were detected using ECL Plus (GE Healthcare) and processed by Amersham Imager 600. To interrogate histone marks equal aliquots of protein were loaded onto multiple gels from a single loading solution. For total protein analysis, 10 µg of whole cell lysates were loaded into each well followed by the above analyses.

**Synergy analyses**

Cells were treated with DMSO or 4-fold dilutions of GS716054 (0.16μM, 0.65μM, 2.5μM, 10μM) for 6 days alone or with 4 concentrations of Ibrutinib (0.16μM, 0.65μM, 2.5μM, 10μM) for 3 days before cell viability was assessed by Cell Titre-Glo described above. A representative treatment matrix is shown in **Supplement Figure S2d**. Synergy score was determined using the SynergyFinder R package (<https://synergyfinder.fimm.fi/>) as described (10, 19).

**Quantitative RT-PCR**

Total RNA from cell lines was extracted using Qiagen RNeasy kits and quantitated by Nano-drop. cDNA was synthesised using the high-capacity cDNA reverse transcription kit (Thermofisher) and qPCR performed using SYBR Green Supermix (Biorad). Reactions were performed in triplicate and normalised to GAPDH.

**RNA-Seq and analysis**

Drug or DMSO treated MINO, JEKO, UPN-1 cells were analyzed by RNA Seq (Novagene, 20M raw Reads, 6GB raw data, UK). mRNA isolations were prepared using Qiagen mRNA Prep kit, quantity and integrity were determined by RNA high-quality range quBits and Agilent Tapestation (RIN>9.5). ≥ 20μg mRNA from 3 independent experiments were used for sequencing. A duplicate cell pellet harvested from the same treatment groups used for RNA-Seq, was used for protein extraction and H3K4me3 levels were validated by Western blotting.

FASTQ files were aligned to GRCh38 using Hisat2 (version 2.1.0) (20). Reads aligning to exons were counted using htseq-count (version 0.13.5) (21). Differential gene expression (DE) analysis between the drug and DMSO-treated cells was doned for each cell line at each timepoint using the DESeq2 package (version 1.36.0) (22). DE genes were selected based on an adjusted *p*-value of ≤ 0.05.

Gene Set Enrichment Analysis (GSEA) was done using the gene pattern webtool (23), the pre-ranked option and “weighted” scoring method were chosen and the genes ranked by the Wald statistic as determined by DESeq2 (23). We have deposited the RNA-Seq datasets to NCBI under GSE243395.

**Cell-cycle and apoptosis analysis**

Cells (3x10^5^/ml) were seeded in 6 well plates for 24h, followed by treatment with DMSO or KDM5-inh1 GS716054 for 72h. Cells were washed in PBS twice before fixation with ice-cold 70% ethanol, stored at 4°C overnight, followed by staining in PBS containing 50μg/ml propidium iodide and 100μg/ml RNase A (FxCycle PI/RNase Staining kit, F10797, Thermofisher) according to the manufacturer’s instructions. DNA content was then quantified on an Attune flow cytometer and analysed by FlowJo software. Percentage of apoptotic cells was quantified by Annexin-V/PI staining kit (eBioscience Annexin V Apoptosis Detection Kit FITC, 88-8005-72, Thermofisher) and assessed by Attune according to the manufacturer’s protocol. Apoptotic cells were defined as Annexin V+/PI+, early apoptotic as Annexin V+/PI- and nucleated debris as Annexin V-/PI+.

**Data analysis and Availability**

Statistical analysis and plots except RNA-Seq were done with Prism Graphpad 8.0 software. RNA-Seq analyses was done in R 4.2.1. Z scored expression data from a cohort of DLBCL (95), FL (65) and MCL (43) samples was downloaded from cBioPortal: <https://www.cbioportal.org/study/summary?id=mbn_mdacc_2013>. Expression of KDM5A, B, C and D isoforms were compared between lymphoma by Kruskal-Wallis test.

**References**

19. Tong KI, Yoon S, Isaev K, Bakhtiari M, Lackraj T, He MY, Joynt J, Silva A, Xu MC, Privé GG, He HH, Tiedemann RE, Chavez EA, Chong LC, Boyle M, Scott DW, Steidl C, Kridel R. Combined EZH2 Inhibition and IKAROS Degradation Leads to Enhanced Antitumor Activity in Diffuse Large B-cell Lymphoma. Clin Cancer Res. 2021 Oct 1;27(19):5401-5414.

20. Kim, D., Paggi, J.M., Park, C. et al. Graph-based genome alignment and genotyping with HISAT2 and HISAT-genotype. Nat Biotechnol 2019, 37, 907–915.

21. Simon Anders and others, HTSeq—a Python framework to work with high-throughput sequencing data, Bioinformatics 2015 Jan, 31(2), 166–169.

22. Love, M.I., Huber, W. & Anders, S. Moderated estimation of fold change and dispersion for RNA-seq data with DESeq2. Genome Biol 2014, 15, 550.

1. Reich M, Liefeld T, Gould J, Lerner J, Tamayo P, Mesirov JP. GenePattern 2.0. Nat Genet. 2006 May;38(5):500-1.

**Supplementary Figure Legends**

**Figure S1 JQKD82 induces H3K4me3 levels to the same extent of GS716054 but less potent on inducing cytotoxic response on MCL**. **(a)** Cell viability following 6 days exposure to DMSO or 6 dose concentrations of JQKD82 (Dana Farber Cancer Institute) ranging from 0.00031 µM to 10 µM, determined by Cell-Titre Glow assays. **(b)** Bar chart showing the EC50 values of MCL and DLBCL cell lines**. (c)** H3K4me3 levels from cell extracts following treatment as described in **Fig 1g** were measured. Representative H3 level as loading control. **(d)** Z138 and JEKO cells were exposed to DMSO, 1 or 5µM GS716054 for 72h, BCL2 were assessed by comparison with DLBCL cell lines HT and SUDHL-6.

**Figure S2 Sensitivity of MCL cell lines to Ibrutinib was determined and KDM5-inhibitor is synergistic with Ibrutinib in most MCL cell lines.** **(a)** Cell viability following 3 days treatment to DMSO or 6 dose concentrations of Ibrutinib ranging from 0.00031 µM to 10 µM, determined by Cell Titer-Glo assays. **(b)** Bar chart showing the EC_50_ values of Ibrutinib on MCL and DLBCL cell lines**. (c)** MINO cell lines stably expressing either WT BTK or BTK C481S mutant were treated with Ibrutinib as described in **(a)** to determine the sensitivity to Ibrutinib**. (d)** Representative dose dependent matrix showing the inhibition of viable JEKO cells after treatment with increasing dosing of GS716054 for 6 days, alongside increasing concentrations of Ibrutinib for 3 days. (e) Summary of ZIP Synergy score determined in a range of MCL and DLBCL cell lines by at least three independent experiments. Score >10 indicates significant synergy.

**Figure S3 Cell line validation before RNA seq. (a-b)** MINO, JEKO and UPN-1 cells were treated with DMSO or 1µM GS716054 for 24h, 48h, 72h and 144h, cell viability were assessed by Cell Titer-Glo in **(a).** H3K4me3 levels were determined by Western blot by comparison with H3 in **(b)**. **(c)**. MINO, JEKO and UPN-1 cells were treated with DMSO or 1µM GS716054 for 72h, histone marks were assessed from protein extracts by Western Blot, alongside RNA extracts used for sequencing.

**Figure S4 Differential expressed gene sets identified by RNA-Seq following KDM5-inhibition**. **(a)** Bar chart showing the number of differentially upregulated or downregulated genes in indicated drug-treated cells (1µM GS716054 for 24h or 72h) relative to the gene expression in cells treated with DMSO controls. RNA Seq Data were generated from 3 biological triplicates. **(b)** Overlap between GS716054 regulated genes at 24h and 72h in MINO, JEKO and UPN-1 cells. Log2FC>0 indicates up-regulated genes while Log2FC<0 for down-regulated genes.

**Figure S5 Targets following KDM5-inhibition are validated. (a)** A variety of MCL, DLBCL and MM cell lines were treated with DMSO or 1µM GS716054 for 72h, the protein levels of LCK, c-MYC, CyclinD1 and GAPDH, alongside of H3K4me3 and H3, were assessed by Western blot. **(b)** MINO cell lines stably expressing either WT BTK or BTK C481S mutant, were treated with DMSO or 1µM GS716054 for 72 h. LCK, c-MYC expressions were determined.

**Figure S6 Enrichment of MYC target genes following KDM5 inhibition.** GSEA plots showing enrichment of MYC target genes in MINO cells across 24h and 72h time points. Each from three independent RNA samples.

**Figure S7 KDM5 inhibition regulates MCL cell death through cell cycle controls. (a)** JEKO cells were treated with DMSO or increasing dose of GS716054 for 72hrs. Cells were stained with Annexin V and Propidium Iodide, apoptotic cell percentages were analyzed by flow cytometry. **(b)**JEKO cells were treated with DMSO, 1µM GS716054 or 1µM JQKD82 for 72 h. Cell cycle profiles were analyzed by flow cytometry. **(c)** Bubble plot indicates the strength of enrichment (NES) for the cell cycle associated reactome. Size of the bubbles indicates significance.
