# Supplementary figures and images for: Targeting lysine demethylase 5 (KDM5) in mantle cell lymphoma
Source: Blood Cancer J. 2024 Feb 13;14(1):29. doi: 10.1038/s41408-024-00999-8 (PMC10864367; doi:10.1038/s41408-024-00999-8)

Figure S-1

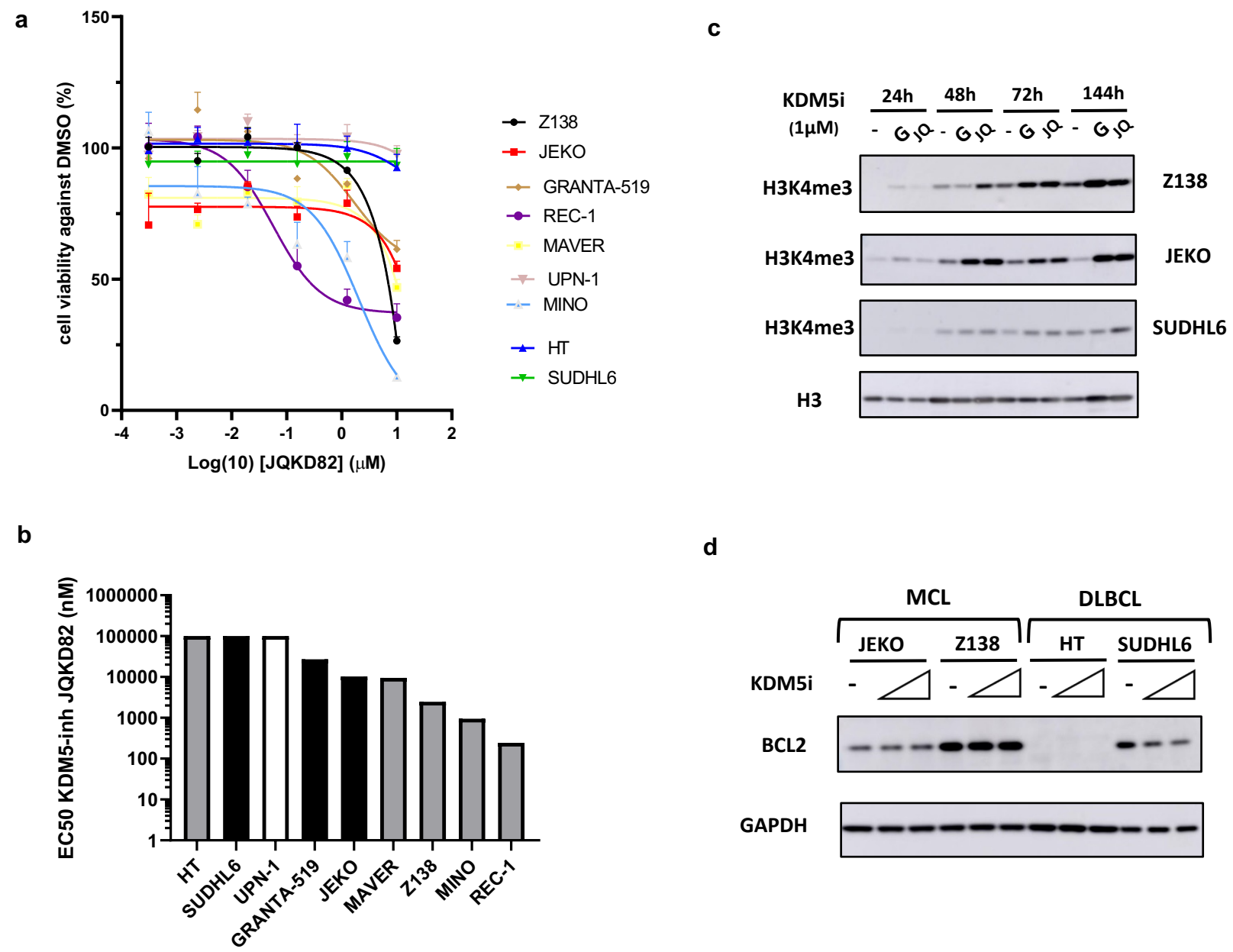

Figure S-2

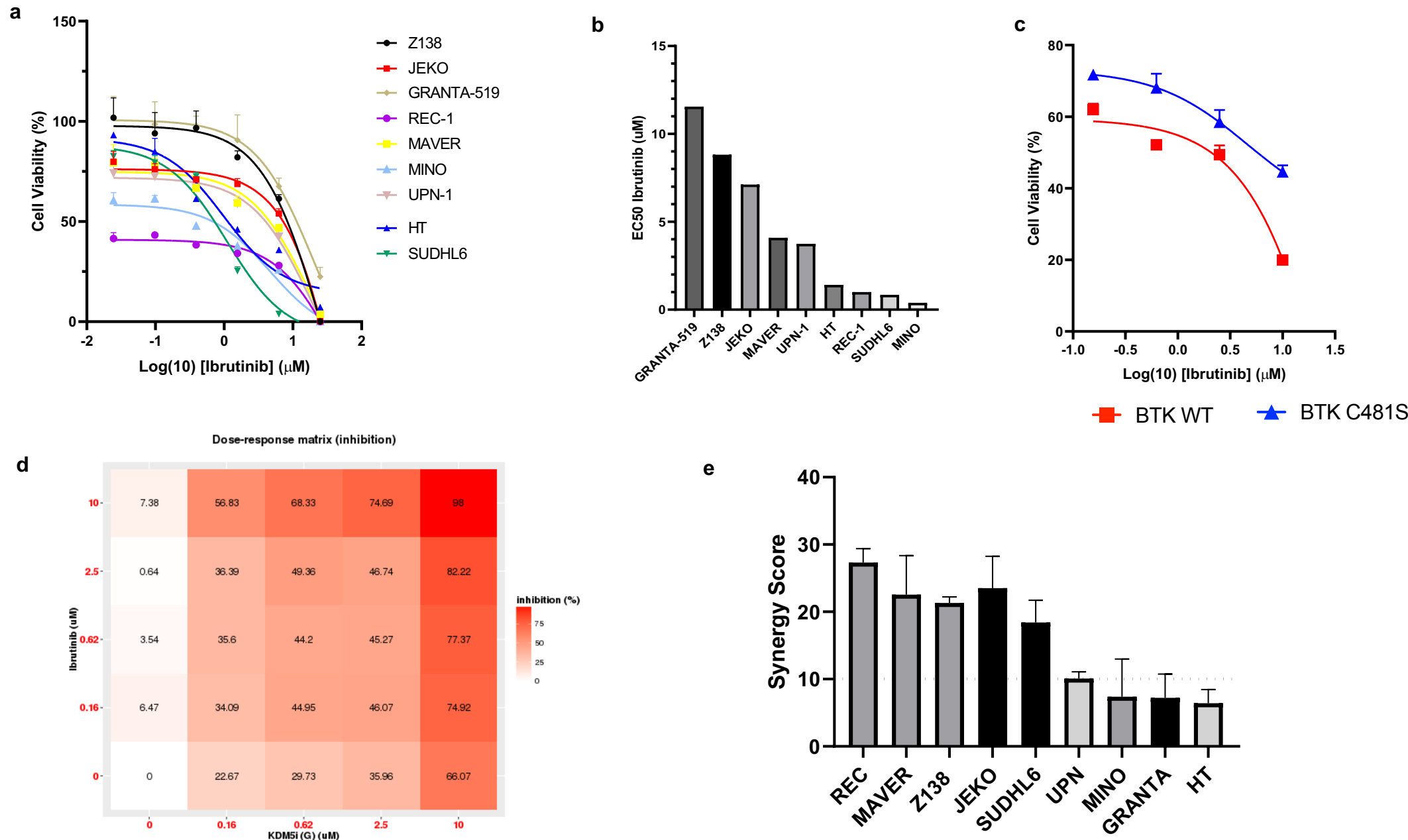

Figure S-3

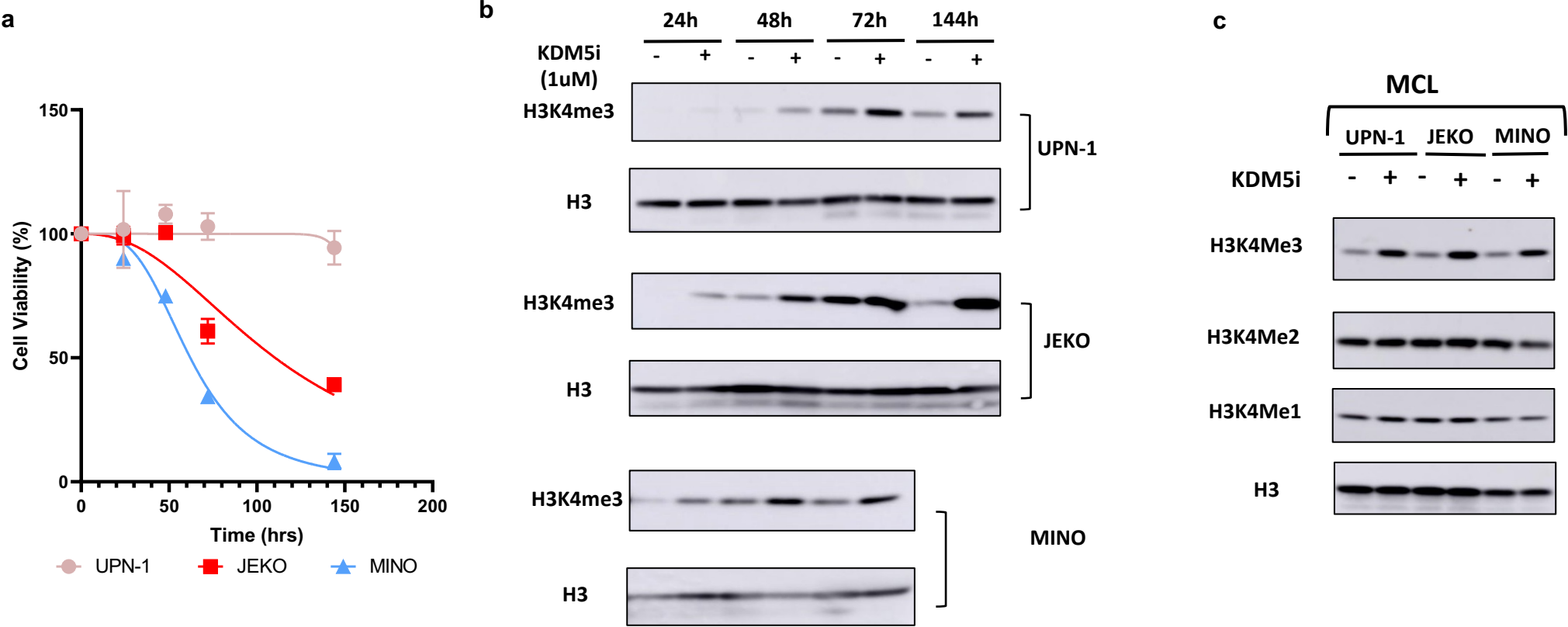

Figure S-4

a

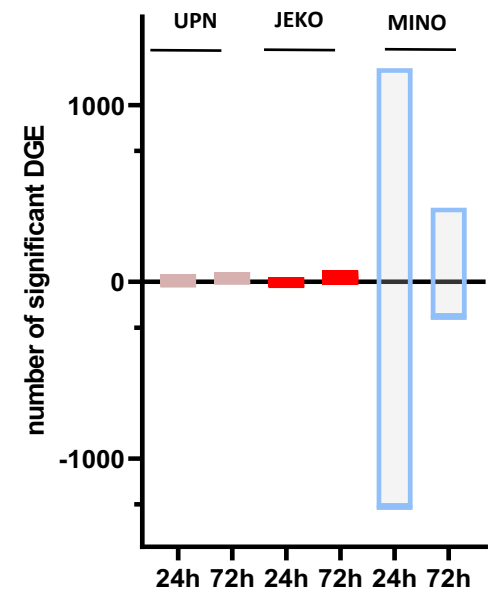

b

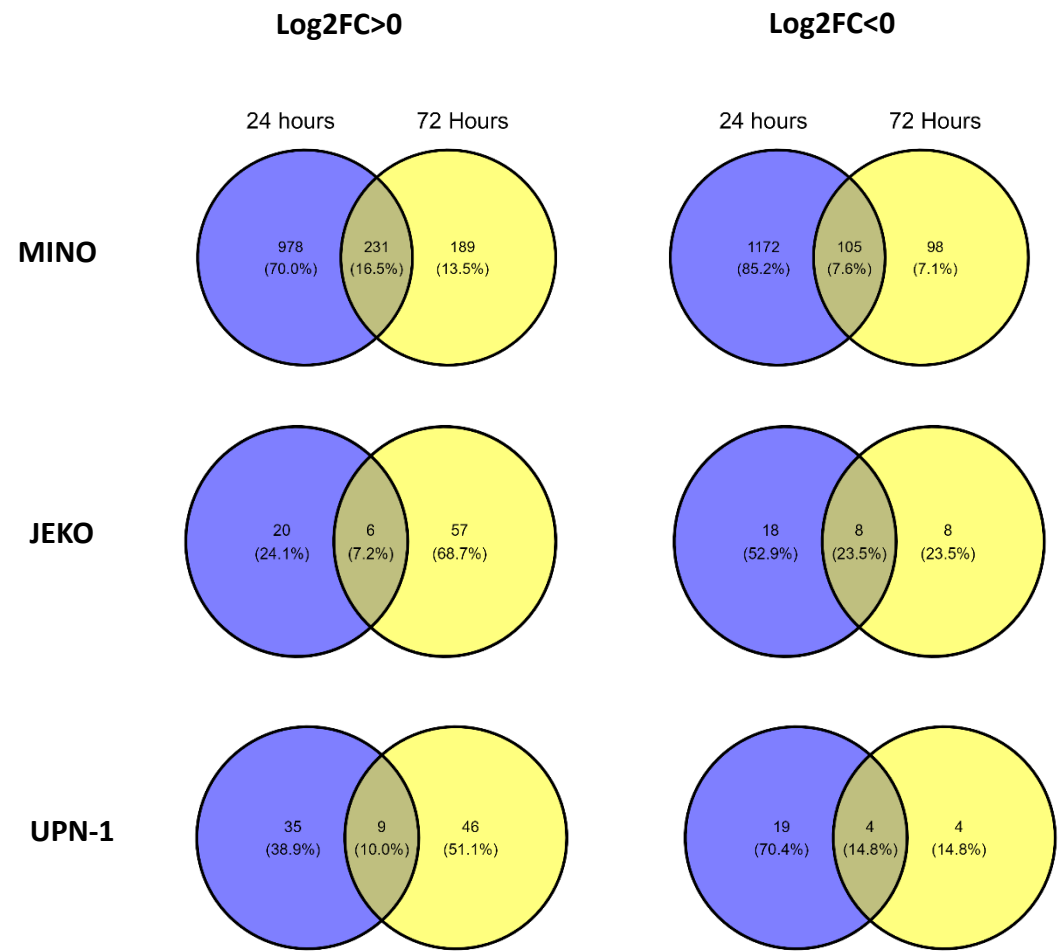

Figure S-5

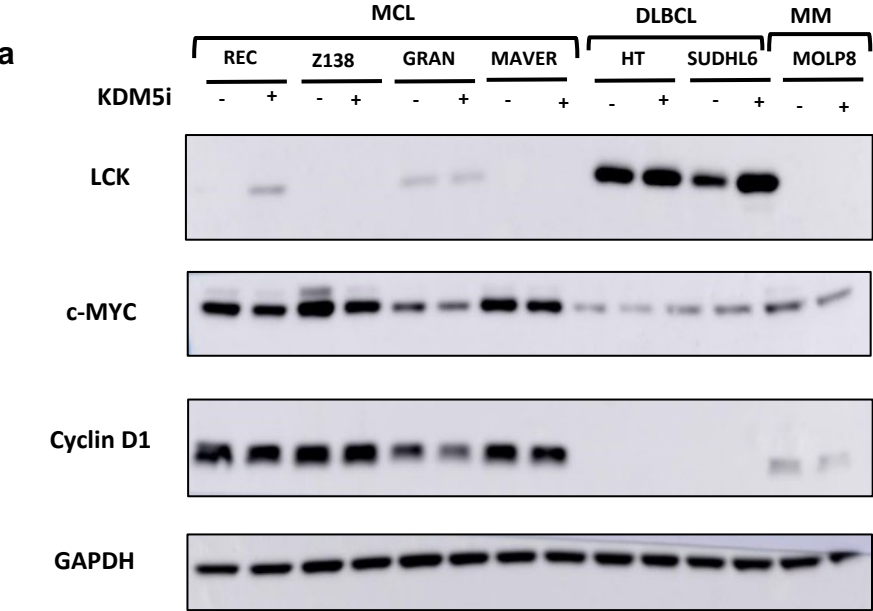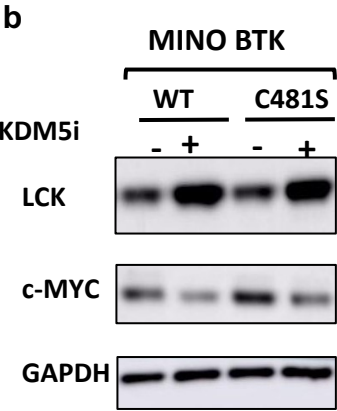

Figure S-6

24h

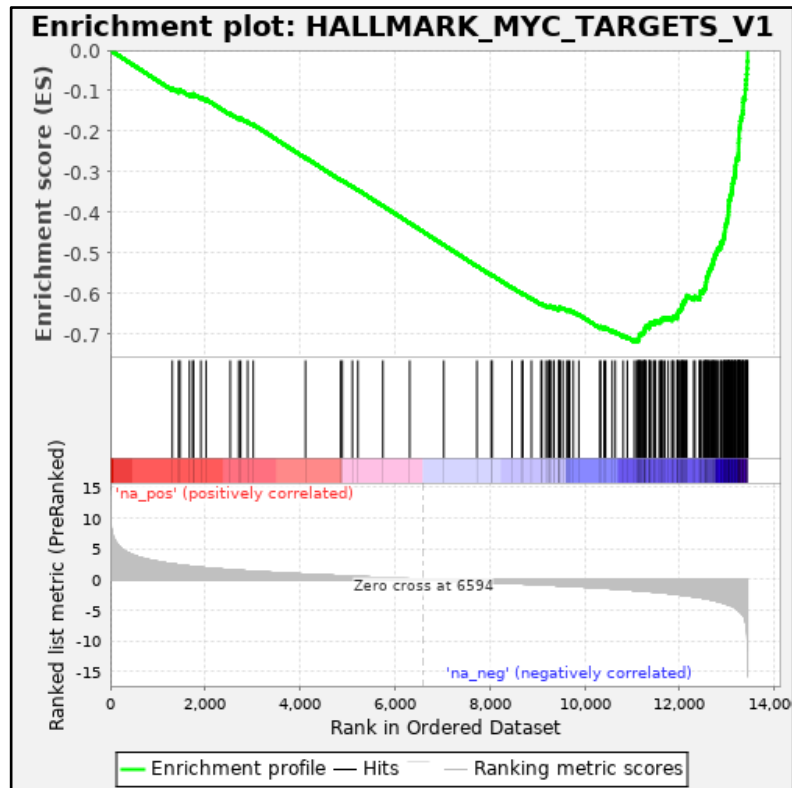

72h

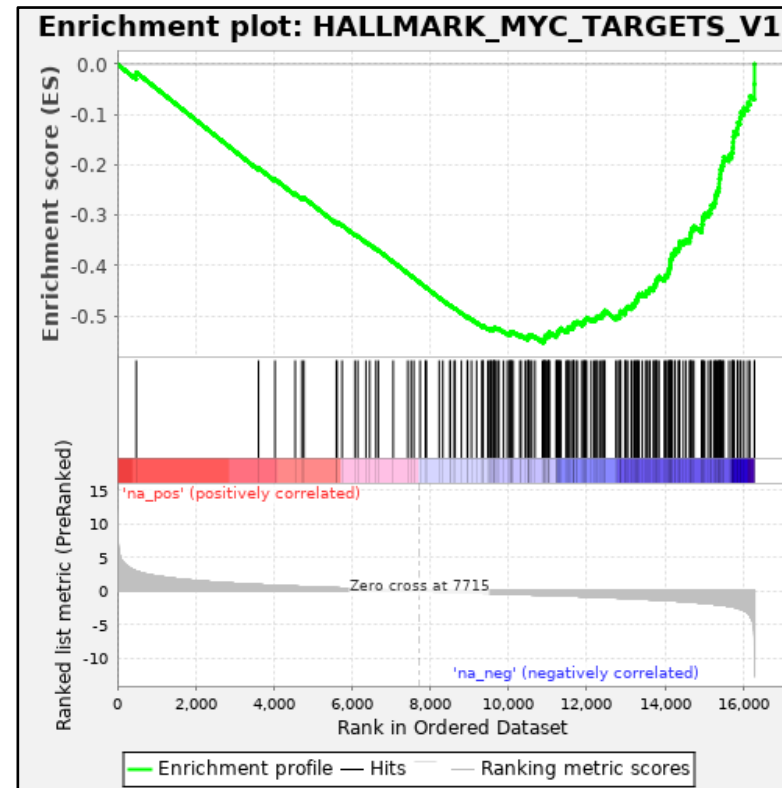

Figure S-7

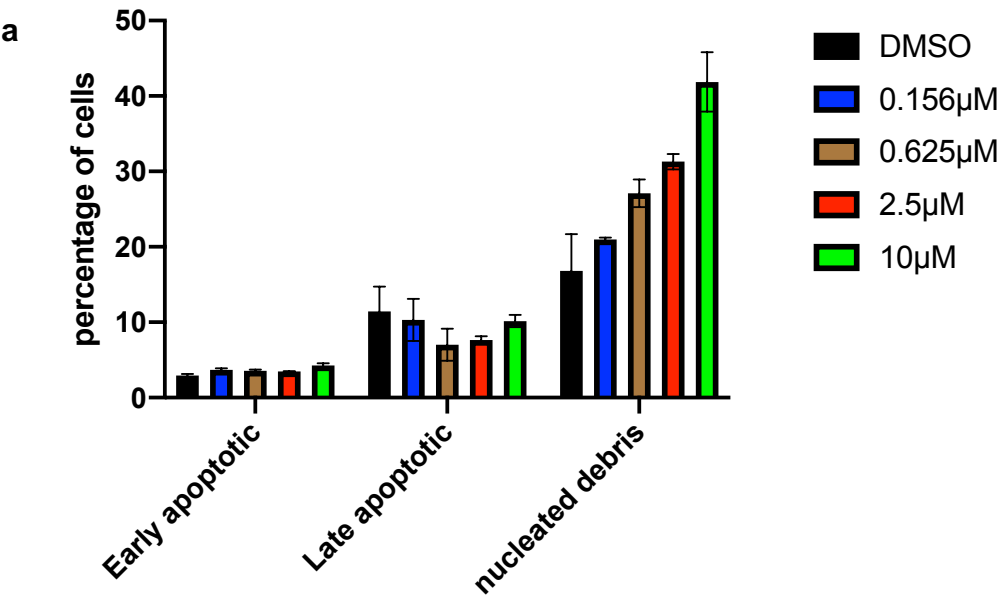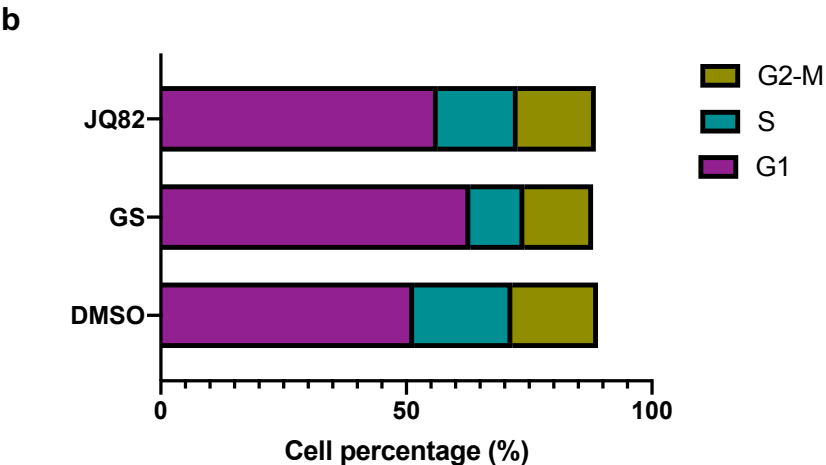

**c**

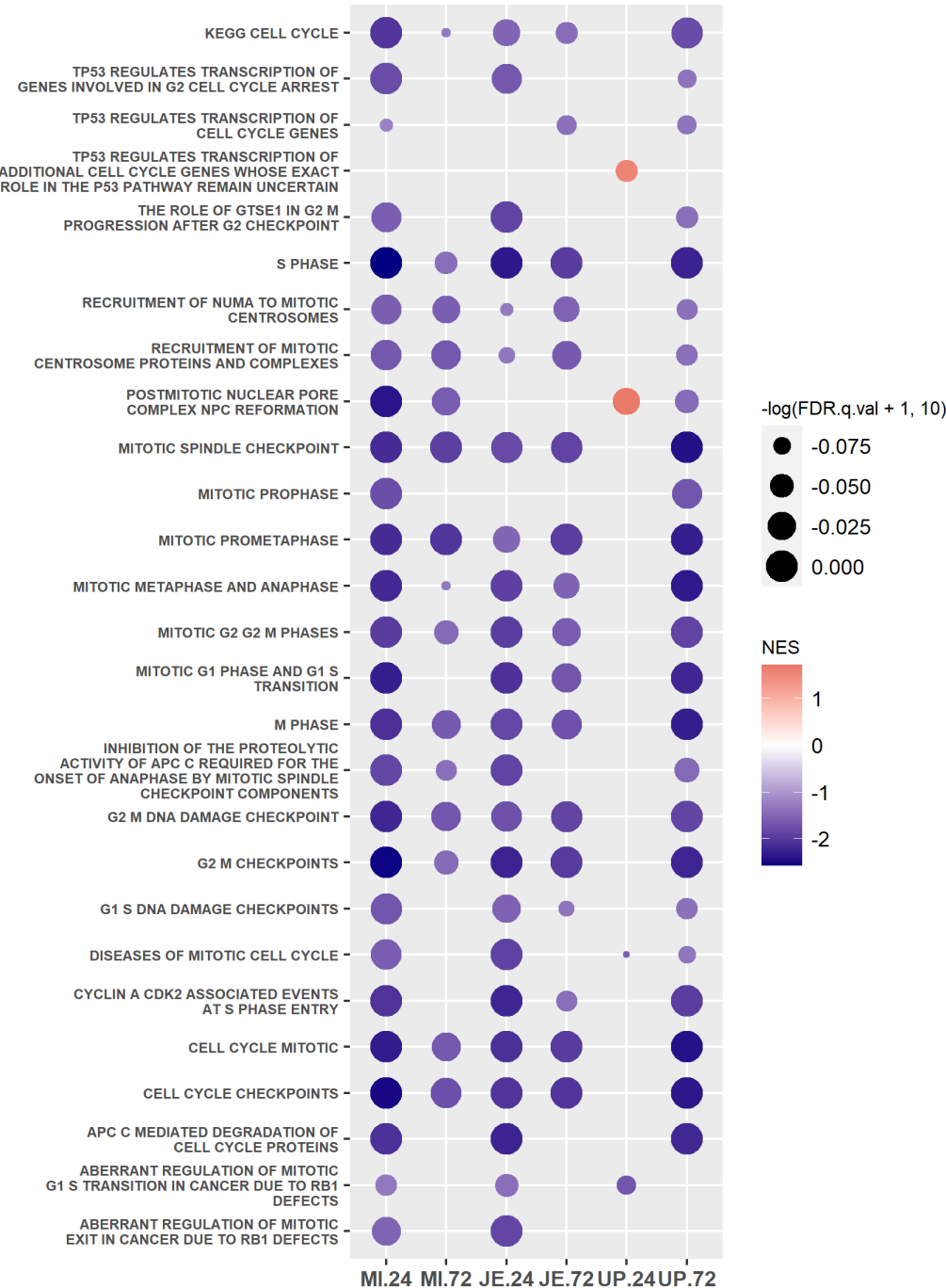

Supplement: Supplementary file 2 — supplementary figures [file 41408_2024_999_MOESM2_ESM.pdf]
