# Supplementary material for: Targeting lysine demethylase 5 (KDM5) in mantle cell lymphoma
Source: Blood Cancer J. 2024 Feb 13;14(1):29. doi: 10.1038/s41408-024-00999-8 (PMC10864367; doi:10.1038/s41408-024-00999-8)
Supplement: Supplementary file 3 — Supplemental Table [file 41408_2024_999_MOESM3_ESM.pdf]

Cell lines	Disease	Culture Medium	KMT2D	p53	Other
JEKO-1	MCL	RPMI1640+20%FBS	R5225C	P58fs	
Granta-519	MCL	DMEM +10%FBS	A1598V	WT	
MINO	MCL	RPMI1640+20%FBS	WT	V417G	
MINO BTK ^{wt}	MCL	RPMI1640+20%FBS	WT	V417G	BTK WT
MINO BTK ^{C481S}	MCL	RPMI1640+20%FBS	WT	V417G	BTK C481S mutant
REC-1	MCL	RPMI1640+20%FBS	WT	Q317*,G245D	
UPN-1	MCL	RPMI1640+10%FBS	NA	NA	
Z-138	MCL	IMEM +10%FBS	WT	WT	
MAVER-1	MCL	RPMI1640+20%FBS	WT	D281E	
HT	GCB-DLBCL	RPMI1640+10%FBS	WT	R273H, V216M	
SUDHL-6	GCB-DLBCL	RPMI1640+20%FBS	E4712*, Q211*	Y234C	
MOLP8	MM	RPMI1640+20%FBS	WT	WT	
